# Oxybutynin for Primary Palmer Hyperhidrosis Attenuates Migraine Attacks and Burdens

**DOI:** 10.7759/cureus.44826

**Published:** 2023-09-07

**Authors:** Masahito Katsuki

**Affiliations:** 1 Neurology, Komuginomori Headache Clinic, Shiojiri, JPN

**Keywords:** anxiety, alternative medical therapies, oxybutynin, japanese herbal kampo medicine, valproic acid, hyperhidrosis, headache

## Abstract

Migraine is a neurological disorder with recurrent headaches accompanied by burdens in social life. Primary palmar hyperhidrosis is a chronic condition with excessive sweating of the palms that can significantly impair quality of life. Primary hyperhidrosis can cause anxiety, and stress, including anxiety, is the most common inducer of migraine headaches. Recently, oxybutynin has been used for primary palmar hyperhidrosis. We herein describe a 26-year-old female migraine patient with primary palmar hyperhidrosis whose migraine attacks and burdens were attenuated after the prescription of an oxybutynin lotion formula. The patient’s monthly headache days (MHD) and monthly acute medication intake days (AMD) at the first visit were 10 and 9. Headache Impact Score 6 (HIT-6) at the initial visit was 63. After the prescription of Japanese herbal kampo medicine Goreisan (TJ-17), Goshuyuto (TJ-31), and 200 mg of valproic acid, MHD, AMD, and HIT-6 decreased gradually. However, these parameters could not improve sufficiently at nine months: MHD 4, AMD 4, and HIT-6 52. We first prescribed a lotion formulation of 20% oxybutynin hydrochloride at nine months. After this, migraine was further attenuated, and stress related to primary palmar hyperhidrosis was reduced; at 12 months, the patient had achieved MHD 2, AMD 2, and HIT-6 48. She will continue receiving primary palmar hyperhidrosis treatment while tapering off migraine prophylaxis. While the exact mechanisms connecting migraine and primary hyperhidrosis remain uncertain, this case raises important questions about the potential interplay between stress, sweating, and migraine triggers.

## Introduction

Migraine is a neurological disorder characterized by recurrent episodes of severe headache, often accompanied by other symptoms such as nausea, vomiting, and sensitivity to light and sound. The epidemiological prevalence in Japan is 8.4%, and those in other countries are higher [1]. Migraine impairs daily life, and not only acute medication for migraine attacks but also prophylactic medications to suppress the intensity and frequency are important [2]. However, it is often difficult for oral prophylactic drugs to provide adequate therapeutic efficacy, and although new drugs such as calcitonin gene-related peptide (CGRP)-related drugs have been introduced [3], control by oral therapy is still important, considering drug costs.

Primary palmar hyperhidrosis is a chronic condition characterized by excessive sweating of the palms that can significantly impair daily activities and quality of life. The epidemiological survey revealed the prevalence of primary palmer hyperhidrosis as 5.22% among the 5- to 64-year-old population [4]. Patients with mild or moderate primary palmar hyperhidrosis are at a higher risk of anxiety induced by sweating than participants [5]. Stress, including anxiety, is the most common inducer of migraine headaches [6,7]. Therefore, if sweating is causing stress, suppressing sweating may also suppress migraine attacks as a secondary effect. Until now, iontophoresis, topical 20-50% aluminum chloride, botulinum toxin-A injection, and sympathetic nerve blockade have been used, but their efficacy was insufficient and the burden on patients was high. Recently, oxybutynin has been used for primary palmar hyperhidrosis and is now widely used in Japan [4].

We herein describe a migraine patient with primary palmar hyperhidrosis whose migraine attacks and burdens were attenuated after the prescription of a lotion formulation of 20% oxybutynin hydrochloride.

## Case presentation

A 26-year-old female presented with a chronic headache on a numeric rating scale of 2/10 to 8/10 as an outpatient, with a long-standing history of primary palmar hyperhidrosis and recurrent migraine attacks. The headache was not pulsatile but unilateral. She had experienced headache aggravation and avoided physical activity due to the headache, and she also felt nausea. The attacks last for about six hours. She had suffered from migraines since age 16, and the frequency and intensity of migraine attacks had worsened over the last three years. She had never consulted a neurologist about her headache or a dermatologist about her palmar hyperhidrosis. She used over-the-counter analgesics as acute medication, and no prophylactic medications had been tried. We diagnosed migraine without aura (the International Classification of Headache Disorders, 3rd edition, code 1.1), and we let her keep a headache diary. The monthly headache days (MHD) and monthly acute medication intake days (AMD) at the first visit were 10 and 9. Headache Impact Score 6 (HIT-6) at the initial visit was 63. The HIT-6 score is obtained from a simple summation of the six items and ranges between 36 and 78, with larger scores reflecting greater impact. Headache impact severity levels can be categorized using score ranges based on the HIT-6 interpretation guide. The four headache impact severity categories are little or no impact (49 or less), some impact (50-55), substantial impact (56-59), and severe impact (60-78) [8].

**Figure 1:**
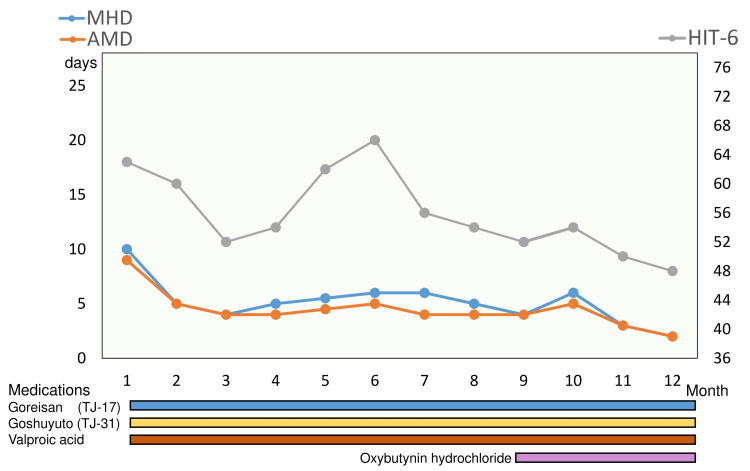
Therapeutic effects Chronological changes of monthly headache days (MHD), monthly acute medication intake days (AMD), and Headache Impact Test 6 (HIT-6) scores after the initial visit. After the prescription of oxybutynin hydrochloride, those three parameters were decreased.

According to the guideline [9], describing that four or more headache days per month are a leading reason for considering preventative therapy, prophylactic medications were started: Japanese herbal kampo medicine Goreisan (TJ-17), Goshuyuto (TJ-31) [10], and 200 mg of valproic acid. The chronological changes of MHD, AMD, and HIT-6 are shown in Figure 1.

After starting prophylactic medications, those parameters gradually decreased. However, at nine months, the patient’s MHD, AMD, and HIT-6 were 4, 4, and 52, respectively. Because of concerns about drowsiness, there was no desire to further increase the dose of valproic acid or add other medications. It was only then, for the first time, that she also asked about palmar hyperhidrosis. She has experienced excessive sweating of her palms since adolescence, which significantly affected her social interactions and self-confidence. We prescribed a lotion formulation of 20% oxybutynin hydrochloride. At 12 months, the patient had achieved MHD 2, AMD 2, and HIT-6 48. MHD and AMD decreased, and HIT-6 was within the normal range as it was below 50. She will continue receiving primary palmar hyperhidrosis treatment while tapering off valproic acid and Japanese herbal kampo medicines. Also, she reported that her palmar hyperhidrosis-induced stress was significantly reduced after the initiation of oxybutynin treatment.

## Discussion

The presented case report describes a patient with primary palmar hyperhidrosis and migraine attacks whose migraine frequency and burdens were attenuated after the prescription of a lotion formulation of 20% oxybutynin hydrochloride. This case raises several interesting points regarding the potential relationship between primary palmar hyperhidrosis, migraine, and the therapeutic use of oxybutynin.

The potential link between hyperhidrosis and migraine

The patient had been experiencing excessive sweating of her palms since adolescence, which might have led to social distress and impaired self-confidence. The relationship between primary palmar hyperhidrosis and migraines is intriguing, especially considering that stress [6,7], including anxiety induced by sweating [5], has been identified as a common trigger for migraine headaches. This link between stress and migraines could suggest that migraine attacks could also be alleviated by suppressing sweating and subsequently reducing stress levels. The patient's primary palmar hyperhidrosis-induced stress was significantly reduced after the initiation of oxybutynin treatment. It is also worth considering that by addressing the patient's psychological distress related to primary palmar hyperhidrosis, the overall emotional well-being improved, potentially influencing migraine triggers.

A recent investigation has revealed that individuals suffering from migraines exhibit heightened sensitivity to wetness, particularly when exposed to cold and wet stimuli on their foreheads, as opposed to healthy individuals. This sensitivity to cold and wet conditions could potentially be attributed to the involvement of the transient receptor potential cation channel subfamily M (melastatin) member 8 (TRPM8) [11]. This channel is known to be activated by various factors, such as moisture, cold stimuli, and chemicals. It notably stands out as the singular temperature receptor responsible for eliciting an inherent response to mild cold and moisture. Given this heightened sensitivity, individuals with migraines may demonstrate an amplified response to damp and chilly stimuli, such as humidity and rain. These external environmental stressors could potentially impact or modify the hypothalamus, which might serve as a trigger for migraine episodes through its interactions with the hypothalamus [12]. If a migraine patient presents with primary palmar hyperhidrosis, the wet or cold stimulus may itself be a migraine trigger.

Oxybutynin's potential mechanism of action

Oxybutynin is an anticholinergic medication [4] that has been traditionally used to manage overactive bladder and urinary incontinence by reducing muscle spasms in the bladder. However, it has also been found to effectively manage primary palmar hyperhidrosis by inhibiting sweat production. The mechanism by which oxybutynin exerts its effects on both primary palmar hyperhidrosis and migraine remains speculative.

In addition to CGRP, novel therapeutic targets have been found for migraine [13]. Acetylcholine (ACh) is a classic neurotransmitter, but it has also been linked to the pathogenesis of migraine. ACh is likely responsible for triggering the release of mast cell contents, a process known as degranulation, potentially through muscarinic ACh receptors, particularly the M3 subtype. Additionally, ACh activation could lead to the stimulation of trigeminal nerve fibers via specific ACh receptors. Previous research indicates that the activation of parasympathetic nerve fibers has been associated with events like the leakage of plasma from venous blood vessels and an increase in blood flow in the meninges. This increased blood flow is thought to be driven by the dilation of arterial vessels. This dilation could possibly be influenced by endothelial ACh receptors, particularly the M3 subtype, which signal through nitric oxide pathways [14]. The oxybutynin component absorbed through the skin may have reduced the intensity and frequency of migraine headaches by inhibiting parasympathetic nerves to the trigeminal nerve, thereby preventing vasodilation.

It has also been shown that migraine patients have reduced sympathetic activity during the interictal phase of attacks. Therefore, it is possible that oxybutynin may have normalized autonomic nervous system activity by inhibiting acetylcholine and suppressing the parasympathetic nervous system [15], thereby activating the sympathetic nervous system relatively. On the other hand, patients with primary palmar hyperhidrosis often have increased sympathetic activity [4,5], which is inconsistent with the fact that sympathetic activity is decreased in migraine patients during the interictal phase. Further study of the autonomic nervous system, migraine, and primary palmar hyperhidrosis is warranted. Furthermore, oxybutynin’s relationship to neurotransmission and/or Ca2+ flux effects should be investigated.

Limitations and considerations

As a limitation, we did not examine how much stress was directly due to hyperhidrosis, such as with a stress check test. The patient initially received prophylactic medications for her migraines, which led to a gradual reduction in migraine frequency and burden. However, a complete resolution was not achieved, and concerns about drowsiness limited the ability to further increase the dose of the prophylactic medication. It was only after addressing the primary palmar hyperhidrosis with oxybutynin that the patient achieved a substantial improvement in both her migraine symptoms and her palmar hyperhidrosis. This suggests the possibility of a synergistic effect when treating both conditions simultaneously. While adequate prophylactic medications have not yet been developed for migraine, it may be a good idea to do what may improve the situation, even if only slightly.

## Conclusions

The presented case highlights a unique observation of a patient with primary palmar hyperhidrosis and migraines experiencing significant improvements in migraine frequency and burdens following the initiation of oxybutynin treatment for primary palmar hyperhidrosis. While the exact mechanisms connecting these conditions remain uncertain, this case raises important questions about the potential interplay between stress, sweating, and migraine triggers. Further research is needed to elucidate the underlying mechanisms and explore the potential benefits of combined therapeutic strategies for patients with coexisting primary palmar hyperhidrosis and migraines.
